# Aging, obesity, and post-therapy cognitive recovery in breast cancer survivors

**DOI:** 10.18632/oncotarget.12565

**Published:** 2016-10-11

**Authors:** Zhezhou Huang, Ying Zheng, Pingping Bao, Hui Cai, Zhen Hong, Ding Ding, James Jackson, Xiao-Ou Shu, Qi Dai

**Affiliations:** ^1^ Department of Elderly Health, Division of Noncommunicable Disease and Injury, Shanghai Municipal Center for Disease Control and Prevention, Shanghai, China; ^2^ Department of Cancer Control and Prevention, Division of Noncommunicable Disease and Injury, Shanghai Municipal Center for Disease Control and Prevention, Shanghai, China; ^3^ Department of Noncommunicable Disease Surveillance, Division of Noncommunicable Disease and Injury, Shanghai Municipal Center for Disease Control and Prevention, Shanghai, China; ^4^ Department of Medicine, Division of Epidemiology, Vanderbilt University School of Medicine, Nashville, Tennessee, USA; ^5^ Department of Neurology, Huashan Hospital, Fudan University, Shanghai, China; ^6^ Division of Allergy, Pulmonary, and Critical Care Medicine, Department of Medicine, Vanderbilt University School of Medicine, Nashville, Tennessee, USA

**Keywords:** breast cancer survivor, cognitive, age, obesity, physical activity

## Abstract

Therapy-induced cognitive impairment is prevalent and long-lasting in cancer survivors, but factors affecting post-therapy cognitive recovery are unclear. We conducted this study to evaluate the associations of age, body mass index (BMI), waist-to-hip ratio (WHR), and physical activity (PA) with post-therapy cognitive changes in a population-based breast cancer (BC) survivor cohort. We collected information on PA, weight, height, waist and hip circumferences of 1286 BC survivors aged 20-75. We assessed their cognitive functions, including immediate memory, delayed memory, verbal fluency, and attention, at 18 and 36 months after cancer diagnosis. Linear regression models were used to examine the associations of age, BMI, WHR and PA with mean changes in cognitive scores from 18- to 36-month follow-up interview. We found that the post-therapy cognitive changes differed by age and obesity status. Verbal fluency and attention improved in younger patients aged <60 and non-abdominally obese patients, but deteriorated in older patients aged =60 (i.e. verbal fluency and attention) and abdominally obese patients (i.e. verbal fluency). Memory improved in all patients, with a smaller improvement in obese patients compared with normal-weight patients. No significant association was found between PA and post-therapy cognitive change. Due to the novelty of our findings and the limitations of our study, further research, including intervention trials, is warranted to confirm the causal relationship between obesity and cognitive impairments.

## INTRODUCTION

Clinically significant cognitive impairment caused by cancer adjuvant therapy, particularly chemotherapy, is highly prevalent in cancer survivors [1, 2]. Previous prospective studies, including our own, have shown that the cognitive impairment starts to dissipate after the cessation of chemotherapy [3, 4]. However, it persists in some cancer survivors for fully 20 years [5]. Thus, identification of modifiable factors affecting the recovery from cognitive impairment in cancer survivors will benefit this rapidly growing population.

Aging is a well-established risk factor for cognitive decline in the healthy elderly [6]. Previous studies have also linked energy balance-related factors, such as obesity and physical activity (PA), to aging-related cognitive decline [7, 8]. Physiological consequences of obesity such as chronic inflammation, hypertension, hyperlipidemia, insulin resistance and subsequent diabetes, have deleterious effects on innate immunity, which in turn directly harm the central nervous system [9]. PA, a factor interrelated with body fat, may help maintain cognitive function in elders, through enhancing cardiovascular health, helping decrease brain dysfunction and neurodegeneration caused by inflammation, lowering amyloid loading, increasing levels of neurotransmitters and insulin-like growth factor I, etc [10].

However, the associations between energy balance-related factors and post-therapy cognitive recovery in cancer survivors have not been investigated. We hypothesized that energy balance-related factors affect the post-therapy cognitive recovery in cancer survivors. Hence, we conducted this study to longitudinally investigate the associations of age and energy balance-related factors, including body mass index (BMI), waist-to-hip ratio (WHR, i.e. a measure of visceral adiposity) and PA, with post-therapy cognitive recovery in a large cohort of breast cancer (BC) survivors.

## RESULTS

As shown in Table 1, we found no significant differences in demographic characteristics, menopausal status, depression, cancer diagnosis, progression and treatments, time since the cessation of cancer therapy, BMI, WHR and PA between participants who were eligible for the Cognition Sub-study and who completed the first cognitive assessment, nor between participants who completed the first and second cognitive assessments. Participants included in the Cognition Sub-study had a mean age of 54.9 at the 18-month follow-up interview. Among them, 37.3% were overweight (24.0 ≤ BMI < 28.0) and 13.4% were obese (BMI ≥ 28.0); 47.0% were abdominally obese (WHR ≥ 0.85); and 46.8% were physically inactive (PA intensity < 8.3 METs-hours/week). The majority of our participants (92.9%) underwent chemotherapy. The interval from the cessation of cancer therapy to the 18- and 36-month follow-up interviews was 14.0 and 32.5 months, respectively. The main comorbidities reported by our participants included chronic liver disease (39.1%), hypertension (28.0%), cardiovascular disease (25.5%), chronic gastric disease (20.0%), and diabetes mellitus (12.4%).

**Table 1:** Comparisons of characteristics at 18-month follow-up interview between participants eligible for the Cognition Sub-study and those who completed the study

	Participants eligible for the Cognition Sub-study (*n*=1385)	Participants completed the first cognitive assessment (*n*=1286)	*P*^a^	Participants completed the second cognitive assessment (*n*=1047)	*P*^b^
	Mean (SD) / *n* (%)^c^	Mean (SD) / *n* (%)^c^		Mean (SD) / *n* (%)^c^	
Age at diagnosis (years)	53.4 (9.7)	53.4 (9.7)	0.81	53.5 (9.6)	0.65
Age at 18-month follow-up interview (years)	55.0 (9.7)	54.9 (9.7)	0.81	55.1 (9.6)	0.66
Monthly income (¥/month/capita)			0.97		0.97
<1000	651 (47.0)	600 (46.7)		494 (47.2)	
1000-2000	532 (38.4)	500 (38.9)		402 (38.4)	
> 2000	202 (14.6)	186 (14.4)		151 (14.4)	
Educational attainment			0.78		0.96
Elementary school or below	625 (45.1)	564 (43.8)		464 (44.3)	
Middle or high school	527 (38.1)	505 (39.3)		410 (39.2)	
College or above	233 (16.8)	217 (16.9)		173 (16.5)	
Postmenopausal	1031 (74.4)	956 (74.3)	0.95	786 (75.1)	0.69
Depression score			0.97		0.98
0	427 (40.4)	410 (40.9)		371 (40.9)	
1	378 (35.7)	354 (35.3)		323 (35.6)	
≥2	253 (23.9)	239 (23.8)		213 (23.5)	
Stage of cancer (TNM classification)			1.00		0.98
I	520 (37.6)	488 (38.0)		403 (38.5)	
IIA	479 (34.6)	440 (34.2)		363 (34.7)	
IIB	190 (13.7)	180 (14.0)		146 (13.9)	
III and above	129 (9.3)	116 (9.0)		87 (8.3)	
Unknown	67 (4.8)	62 (4.8)		48 (4.6)	
Ever had surgery	1379 (99.6)	1280 (99.5)	0.90	1042 (99.5)	0.97
Ever undergone radiotherapy	460 (33.2)	428 (33.3)	0.97	345 (33.0)	0.87
Ever undergone chemotherapy	1283 (92.6)	1194 (92.9)	0.83	975 (93.1)	0.79
Ever used tomoxifen	643 (46.5)	595 (46.3)	0.94	497 (47.5)	0.57
Time since the cessation of cancer therapy at 18-month follow-up interview (months)	14.1 (1.9)	14.0 (1.9)	0.81	14.0 (1.9)	0.67
Time since the cessation of cancer therapy at 36-month follow-up interview (months)	32.5 (1.9)	32.5 (1.9)	0.84	32.5 (1.9)	0.57
Breast cancer recurrence or diagnosis of other malignancies after diagnosis of breast cancer	215 (16.3)	194 (15.8)	0.73	146 (14.6)	0.43
Comorbidities					
Cardiovascular disease	302 (25.4)	284 (25.3)	0.92	263 (25.5)	0.91
Hypertension	339 (28.4)	320 (28.2)	0.95	291 (28.0)	0.89
Diabetes mellitus	143 (12.1)	138 (12.3)	0.86	127 (12.4)	0.96
Chronic liver disease	476 (39.7)	448 (39.5)	0.91	407 (39.1)	0.87
Chronic gastric disease	227 (19.0)	217 (19.1)	0.91	206 (20.0)	0.68
Urinary system disease	126 (10.5)	118 (10.4)	0.92	108 (10.4)	0.99
Chronic lung disease	96 (8.0)	93 (8.2)	0.87	87 (8.4)	0.88
BMI (kg/m^2^)			1.00		0.97
Underweight (<18.5)	27 (2.0)	26 (2.0)		20 (1.9)	
Normal (18.5-23.9)	653 (47.1)	608 (47.3)		488 (46.6)	
Overweight (24.0-27.9)	516 (37.3)	480 (37.3)		401 (38.3)	
Obese (≥28.0)	189 (13.6)	172 (13.4)		138 (13.2)	
WHR			0.96		0.84
Normal (<0.80)	297 (21.4)	277 (21.5)		236 (22.6)	
0.80-0.84	430 (31.1)	405 (31.5)		328 (31.3)	
Abdominally obese (≥0.85)	658 (47.5)	604 (47.0)		483 (46.1)	
PA (METs-hours/week)			0.88		0.78
Light-intensity (<8.3)	660 (47.7)	602 (46.8)		477 (45.6)	
Moderate-intensity (8.3-16.5)	306 (22.1)	284 (22.1)		231 (22.0)	
Vigorous-intensity (≥16.6)	419 (30.2)	400 (31.1)		339 (32.4)	

^a^Compared between participants eligible for the Cognition Sub-study and those who completed the first cognitive assessment at 18-month follow-up interview

^b^Compared between participants who completed the first and second cognitive assessments at 18- and 36-month follow-up interviews, respectively

^c^Mean (SD) for continuous variables or *n* (%) for categorical variables

Abbreviations: SD, standard deviation

Table 2 shows the associations of age, BMI, WHR and PA with mean change (MC) in cognitive scores from the 18- to 36-month follow-up interview. Age was significantly associated with mean changes in verbal fluency (*P*^1^ = 0.02) and attention (*P*^1^ < 0.001). Noticeably, verbal fluency improved in younger patients (MC (SE): 1.1 (0.5) and 0.9 (0.3) for patients aged < 50 and 50-59, respectively), but deteriorated in older patients aged ≥ 60 (MC (SE): -0.4 (0.4)). The difference in mean changes in verbal fluency between patients aged 50-59 and ≥ 60 was significant (Diff (SE): -1.3 (0.5), *P*^2^ = 0.01), and it remained significant after being corrected for multiple comparisons (*P*^3^ = 0.03). Similarly, attention also improved in younger patients (MC (SE): 2.6 (0.8) and 3.4 (0.5) for patients aged < 50 and 50-59, respectively), but deteriorated in older patients aged ≥ 60 (MC (SE): -2.2 (0.7)). The difference in mean changes in attention between patients aged 50-59 and ≥ 60 was significant (Diff (SE): -5.6 (0.9), *P*^2^ < 0.001), and it remained significant after being corrected for multiple comparisons (*P*^3^ < 0.001). However, Cohen's *d* values for mean changes in verbal fluency and attention related to age were not clinically significant (both |*d*| < 0.2). The association between age and delayed memory was marginally significant (*P*^1^ = 0.08). Although delayed memory improved across all age groups (all MC > 0), compared with patients aged 50-59, older patients aged ≥ 60 had a smaller improvement (Diff (SE): -0.6 (0.3), *P*^2^ = 0.03). However, this difference was only of borderline significance after being corrected for multiple comparisons (*P*^3^ = 0.08). Overall, BMI was significantly associated with mean change in delayed memory (*P*^1^ = 0.03). Although delayed memory improved across all BMI groups (all MC > 0), compared with normal-weight patients, obese patients (BMI ≥ 28.0) had a smaller improvement (Diff (SE): -0.9 (0.4), *P*^2^ = 0.01). However, this difference was only of borderline significance after being corrected for multiple comparisons (*P*^3^ = 0.07). Cohen's *d* was 0.4 for the improvement in delayed memory among normal-weight patients, indicating a small to moderate clinical significance. WHR was significantly associated with mean change in verbal fluency (*P*^1^ = 0.002). Verbal fluency improved in non-abdominally obese patients (MC (SE): 1.4 (0.4) and 1.1 (0.4) for patients with a WHR ranged < 0.80 and 0.80-0.84, respectively), but slightly declined in abdominally obese patients (MC (SE): -0.2 (0.3)). The difference in mean changes in verbal fluency between patients with a normal WHR and abdominally obese patients was significant (Diff (SE): -1.6 (0.5), *P*^2^ = 0.002), and it remained significant after being corrected for multiple comparisons (*P*^3^ = 0.006). Cohen's *d* was 0.2 for the improvement in verbal fluency among patients with a normal WHR, indicating a borderline small clinical significance. Finally, PA was not significantly associated with mean changes in any cognitive domains (all *P*^1^ > 0.05). Additional interaction analyses examining the interaction between PA and BMI or WHR indicated non-significant interaction effects between PA and BMI or WHR (both *P* > 0.05, data not shown). In sensitivity analyses of the patients who underwent chemotherapy (*n* = 1,194, data not shown), the difference in mean changes in delayed memory between obese patients and normal-weight patients remained significant after being corrected for multiple comparisons (*P*^3^ = 0.03). No substantial difference was found after additional adjustment for comorbidities (data not shown).

**Table 2:** Associations of age, BMI, WHR and PA with changes in cognitive scores from 18- to 36-month follow-up interview (n=1047)

	Immediate memory score						Delayed memory score						Verbal fluency score						Attention score					
	Absolute value		Relative value			Cohen's *d*	Absolute value		Relative value			Cohen's *d*	Absolute value		Relative value			Cohen's *d*	Absolute value		Relative value			Cohen's *d*
	MC (SE)	*P*^1^	Diff (SE)	*P*^2^	*P*^3^		MC (SE)	*P*^1^	Diff (SE)	*P*^2^	*P*^3^		MC (SE)		Diff (SE)	*P*^2^	*P*^3^		MC (SE)	*P*^1^	Diff (SE)	*P*^2^	*P*^3^	
**Age at 36-month follow-up interview (years)^a^**		0.42						0.08			***P*^1^**									<0.001				
<50	1.3 (0.3)		−0.2 (0.3)	0.64	1.00	0.2	1.5 (0.3)		−0.3 (0.3)	0.31	0.02	0.3	1.1 (0.5)		0.2 (0.6)	0.81	1.00	0.1	2.6 (0.8)		−0.8 (1.0)	0.44	1.00	0.1
50-59	1.4 (0.2)		Reference			0.3	1.8 (0.2)		Reference			0.4	0.9 (0.3)		Reference			0.1	3.4 (0.5)		Reference			0.19
≥60	1.1 (0.2)		−0.3 (0.3)	0.19	0.58	0.2	1.2 (0.2)		−0.6 (0.3)	0.03	0.08	0.3	−0.4 (0.4)		−1.3 (0.5)	0.01	0.03	−0.04	−2.2 (0.7)		−5.6 (0.9)	<0.001	<0.001	−0.1
**BMI (kg/m2)^b^**		0.18						0.03						0.41						0.49				
Underweight (<18.5)	1.5 (0.8)		0.1 (0.8)	0.90	1.00	0.4	0.6 (0.8)		−1.1 (0.8)	0.15	0.93	0.1	1.2 (1.4)		0.7 (1.5)	0.64	1.00	0.2	3.3 (2.4)		1.5 (2.5)	0.55	1.00	0.2
Normal (18.5-23.9)	1.4 (0.2)		Reference			0.3	1.7 (0.2)		Reference			0.4	0.5 (0.3)		Reference			0.1	1.9 (0.5)		Reference			0.1
Overweight (24.0-27.9)	1.3 (0.2)		−0.2 (0.2)	0.50	1.00	0.3	1.7 (0.2)		0.04 (0.2)	0.85	1.00	0.4	0.9 (0.3)		0.4 (0.4)	0.39	1.00	0.1	0.9 (0.6)		−0.9 (0.7)	0.23	1.00	0.1
Obese (≥28.0)	0.7 (0.3)		−0.8 (0.3)	0.03	0.17	0.2	0.8 (0.3)		−0.9 (0.4)	0.01	0.07	0.2	−0.2 (0.6)		−0.7 (0.6)	0.30	1.00	−0.02	0.8 (1.0)		−1.0 (1.1)	0.35	1.00	0.0
**WHR^b^**		0.13						0.71						####						0.68				
Normal (<0.80)	0.9 (0.2)		Reference			0.2	1.4 (0.2)		Reference			0.3	1.4 (0.4)		Reference			0.2	1.1 (0.7)		Reference			0.1
0.80-0.84	1.5 (0.2)		0.6 (0.3)	####	0.14	0.4	1.6 (0.2)		0.2 (0.3)	0.43	1.00	0.4	1.1 (0.4)		−0.3 (0.5)	0.64	1.00	0.1	1.8 (0.6)		0.7 (0.9)	0.43	1.00	0.1
Abdominally obese (≥0.85)	1.3 (0.2)		0.4 (0.3)	0.13	0.39	0.3	1.6 (0.2)		0.2 (0.3)	0.49	1.00	0.4	−0.2 (0.3)		−1.6 (0.5)	0.002	####	−0.03	1.3 (0.5)		0.2 (0.9)	0.86	1.00	0.1
**PA (METs-hours/week)^b^**		0.14						0.27						0.10						0.48				
Light-intensity (<8.3)	1.1 (0.2)		−0.4 (0.3)	0.13	0.39	0.2	1.4 (0.2)		−0.3 (0.3)	0.25	0.75	0.3	0.3 (0.3)		−1.0 (0.5)	0.046	0.14	0.04	1.1 (0.5)		−0.05 (0.9)	0.96	1.00	0.1
Moderate-intensity (8.3-16.5)	1.5 (0.2)		Reference			0.3	1.7 (0.2)		Reference			0.4	1.4 (0.4)		Reference			0.2	1.1 (0.7)		Reference			0.1
Vigorous-intensity (≥16.6)	1.5 (0.2)		0.002 (0.3)	0.99	1.00	0.4	1.7 (0.2)		0.04 (0.3)	0.89	1.00	0.4	0.4 (0.3)		−1.0 (0.6)	0.07	0.20	0.05	2.0 (0.6)		0.8 (0.9)	0.37	1.00	0.1

^a^Adjusted for cognitive scores at 18-month follow-up interview, educational attainment, income, menopausal status at 36-month follow-up interview, depression score, stage of cancer, breast cancer recurrence or diagnosis of other malignancies before 36-month follow-up interview, cancer treatments (including surgery, radiotherapy, chemotherapy and tamoxifen use), and time since the cessation of cancer therapy at 36-month follow-up interview.

^b^Addtionally adjusted for age at 36-month follow-up interview

^1^*P*-value for the overall model significance

^2^*P*-value for the difference in mean changes in cognitive scores from 18- to 36-month follow-up interview between this group and the reference group

^3^*P*-value for the difference in mean changes in cognitive score from 18- to 36-month follow-up interview between this group and the reference group after correcting for multiple comparisons using Bonferroni correction

Abbreviations: MC, mean change in cognitive scores from 18- to 36-month follow-up interview for patients in different groups of age, BMI, WHR and PA intensity; SE, standard error; Diff, difference in mean changes in cognitive score from 18- to 36-month follow-up interview between this group and the reference group

Table 3 presents the associations of age, BMI, WHR and PA with cognitive scores at the 18-month follow-up interview using a cross-sectional design. Overall, the associations are in accord with Table 2. Age was significantly associated with all cognitive domains (all *P*^1^ < 0.01). Compared with patients aged 50-59, older patients aged ≥ 60 had significant lower scores in all cognitive domains (all Diff < 0 and *P*^2^ < 0.01), especially in attention (Diff (SE): -16.3 (1.4)). BMI was negatively associated with both delayed memory (*P*^1^ = 0.04) and attention (*P*^1^ < 0.001). WHR was negatively associated with immediate memory (*P*^1^ = 0.002), delayed memory (*P*^1^ = 0.002) and attention (*P*^1^ = 0.003). No significant association was found between PA and cognitive scores at the 18-month follow-up interview (all *P*^1^ > 0.05). No substantial difference was observed in sensitivity analyses of the patients who underwent chemotherapy, nor after additional adjustment for comorbidities.

**Table 3:** Associations of age, BMI, WHR and PA with cognitive scores at 18-month follow-up interview (n=1286)

	Immediate memory score					Delayed memory score					Verbal fluency score					Attention score				
	Absolute value		Relative value			Absolute value		Relative value			Absolute value		Relative value			Absolute value		Relative value		
	Mean (SE)	*P*^1^	Diff (SE)	*P*^2^	*P*^3^	Mean (SE)	*P*^1^	Diff (SE)	*P*^2^	*P*^3^	Mean (SE)	*P*^1^	Diff (SE)	*P*^2^	*P*^3^	Mean (SE)	*P*^1^	Diff (SE)	*P*^2^	*P*^3^
**Age at 18-month follow-up interview (years)^a^**		0.002					0.003					<0.001					<0.001			
<50	9.9 (0.3)		−1.0 (0.4)	0.02	0.0498	9.1 (0.3)		−0.9 (0.4)	0.03	0.08	42.1 (0.5)		−0.9 (0.7)	0.24	0.71	80.6 (1.1)		−1.3 (1.6)	0.42	1.00
50-59	10.8 (0.2)		Reference			10.0 (0.2)		Reference			43.0 (0.4)		Reference			81.9 (0.9)		Reference		
≥60	9.7 (0.3)		−1.2 (0.4)	0.001	0.004	8.9 (0.3)		−1.1 (0.4)	0.002	0.006	40.2 (0.5)		−2.8 (0.7)	<0.001	<0.001	65.5 (1.1)		−16.3 (1.4)	<0.001	<0.001
**BMI (kg/m^2^)^b^**		0.08					0.04					0.23					<0.001			
Underweight (<18.5)	10.2 (1.1)		−0.3 (1.1)	0.78	1.00	10.1 (1.0)		0.3 (1.1)	0.74	1.00	40.9 (1.9)		−1.5 (1.9)	0.45	1.00	80.8 (4.0)		1.9 (4.1)	0.64	1.00
Normal (18.5-23.9)	10.5 (0.2)		Reference			9.8 (0.2)		Reference			42.4 (0.4)		Reference			78.9 (0.8)		Reference		
Overweight (24.0-27.9)	10.1 (0.2)		−0.4 (0.3)	0.17	0.99	9.2 (0.2)		−0.5 (0.3)	0.07	0.42	41.8 (0.4)		−0.6 (0.5)	0.30	1.00	76.3 (0.9)		−2.6 (1.2)	0.03	0.15
Obese (≥28.0)	9.4 (0.4)		−1.1 (0.4)	0.01	0.07	8.6 (0.4)		−1.1 (0.4)	0.010	0.06	40.9 (0.7)		−1.5 (0.8)	0.05	0.32	71.8 (1.5)		−7.1 (1.7)	<0.001	<0.001
**WHR^b^**		0.002					0.002					0.55					0.003			
Normal (<0.80)	10.7 (0.3)		Reference			9.9 (0.3)		Reference			41.5 (0.5)		Reference			78.9 (1.1)		Reference		
0.80-0.84	10.6 (0.2)		−0.04 (0.4)	0.92	1.00	9.8 (0.2)		−0.1 (0.4)	0.85	1.00	42.3 (0.4)		0.7 (0.7)	0.27	0.81	78.7 (0.9)		−0.2 (1.4)	0.89	1.00
Abdominally obese (≥0.85)	9.6 (0.2)		−1.0 (0.4)	0.004	0.01	8.9 (0.2)		−1.0 (0.4)	0.004	0.01	42.0 (0.4)		0.4 (0.7)	0.51	1.00	75.0 (0.8)		−3.8 (1.4)	0.006	0.02
**PA (METs-hours/week)^b^**		0.74					0.41					0.09					0.16			
Light-intensity (<8.3)	10.1 (0.2)		−0.3 (0.4)	0.44	1.00	9.3 (0.2)		−0.5 (0.4)	0.18	0.55	41.5 (0.4)		−0.5 (0.6)	0.47	1.00	76.3 (0.8)		−2.6 (1.4)	0.06	0.17
Moderate-intensity (8.3-16.5)	10.4 (0.3)		Reference			9.7 (0.3)		Reference			41.9 (0.5)		Reference			78.9 (1.1)		Reference		
Vigorous-intensity (≥16.6)	10.2 (0.2)		−0.2 (0.4)	0.56	1.00	9.4 (0.2)		−0.3 (0.4)	0.37	1.00	42.7 (0.4)		0.8 (0.7)	0.24	0.73	76.8 (0.9)		−2.1 (1.4)	0.15	0.46

^a^Adjusted for educational attainment, income, menopausal status at 18-month follow-up interview, depression score, stage of cancer, breast cancer recurrence or diagnosis of other malignancies before 18-month follow-up interview, cancer treatments (including surgery, radiotherapy, chemotherapy and tamoxifen use), and time since the cessation of cancer therapy at 18-month follow-up interview.

^b^Addtionally adjusted for age at 18-month follow-up interview

^1^*P*-value for the overall model significance

^2^*P*-value for the difference in mean cognitive scores at 18-month follow-up interview between this group and the reference group

^3^*P*-value for the difference in mean cognitive scores at 18-month follow-up interview between this group and the reference group after correcting for multiple comparisons using Bonferroni correction

Abbreviations: Mean, mean cognitive score at 18-month follow-up interview for patients in different groups of age, BMI, WHR and PA intensity; SE, standard error; Diff, difference in mean cognitive scores at 18-month follow-up interview between this group and the reference group

## DISCUSSION

To the best of our knowledge, this is the first study to evaluate the associations of energy balance related factors (i.e. BMI, WHR and PA) and age with post-therapy cognitive recovery in BC survivors. We found that verbal fluency and attention improved in younger patients aged < 60 years and non-abdominally obese patients from 18 to 36 months after cancer diagnosis, but deteriorated among older patients aged ≥ 60 years (i.e. verbal fluency and attention) and abdominally obese patients (i.e. verbal fluency); the deterioration in attention was more substantial than that in verbal fluency for older patients. Memory improved in all patients; however, compared with normal-weight patients, the improvement was significantly smaller in obese patients. Normal BMI indicated a small to moderate clinical significance on memory recovery, and normal WHR indicated a borderline small clinical significance on verbal fluency recovery. No significant association was found between PA and post-therapy cognitive recovery.

Our finding that the post-therapy cognitive recovery differed by age is consistent with biological theories of aging [11–13]. Normal aging in human leads to disproportionate structural changes in brain and variable degrees of cognitive decline in later life [13]. Although the relationship between brain atrophy and cognition decline needs to be continuously explored, structural imaging of the brain has recently provided an unprecedented view of age-related differences in structural brain measures and cognitive performance across a broad range of cognitive domains [14]. For example, executive functions involving attention, processing speed and inhibitory control, rely heavily, although not exclusively, on the frontal cortex. The volume and function of frontal cortex show three phases of atrophy. Its atrophy rate accelerates from age 20 to 40, slows down between 40 and 60, and accelerates again thereafter [12, 14]. Moreover, memory formation depends on the hippocampus, which has a stable rate of atrophy until age 60, and the rate accelerates substantially with advancing age thereafter [14–16]. The opposite directions of longitudinal changes in verbal fluency and attention observed in patients aged < 60 and ≥ 60 are consistent with the pattern of frontal cortex atrophy. It is logical to suspect that patients aged ≥ 60 were at an accelerated rate of frontal cortex atrophy, and their aging effect surpassed their recovery from therapy-induced cognitive impairment. The aging effect in patients aged < 60 who were at a lower rate of frontal cortex atrophy, might not be large enough to counteract their post-therapy cognitive recovery. Further, older patients have lower cognitive reserve at pretreatment, which may be caused by underlying genetic factors as well as polypharmacy [17, 18]. Therefore, they are likely to be more vulnerable to therapy-induced cognitive impairment. Future studies are needed to confirm our finding on the age-related discrepancy in post-therapy cognitive recovery.

Obesity is linked to cognitive impairment in the healthy elderly [10, 19]. The underlying physiological mechanisms are complex and may involve several possibilities, including obesity-associated activation of innate immunity and structural brain change. Increased body mass index (BMI) is significantly related to shorter neuronal fiber bundle length and reduced brain volume, resulting in cognitive decline [20–24]. Recent evidence also suggests that WHR is independently associated with cognitive decline [25–27]. A cross-sectional study on central obesity and structural magnetic resonance imaging reported that greater WHR was associated with smaller hippocampi and more white matter hyperintensities after being adjusted for BMI and other risk factors [25]. Epidemiologic evidence linking obesity to cancer risk is convincing for several cancers, including postmenopausal breast cancer. Identified biological mechanisms that explain this link include hormonal alterations (eg, estrogen and leptin), induction of insulin-signaling pathways, activation of proinflammatory pathways, etc [28]. Furthermore, obesity appears to be a negative prognostic factor for recurrence and survival among both premenopausal and postmenopausal women with breast cancer [28]. However, some recent studies challenge this by demonstrating that overweight and early obese states are associated with improved survival, known as “obesity paradox” [29, 30]. It remains controversial whether the “obesity paradox” is a real association or an artificial association due to confounding and bias [30]. Our study found the post-therapy cognitive recovery in BC survivors also differed by obesity status. Normal-weight and overweight patients had the greatest improvements in memory. There is a profound implication of our finding that verbal fluency deteriorated in abdominally obese patients. Due to the novelty of our findings and the limitations of this study, further research, including intervention trials, is warranted to confirm the causal relationship between obesity and cognitive impairments.

Growing evidence suggests that exercise is related to better cognitive function in BC survivors [31–35], however, the relationship may be dose-dependent (intensity and duration) in the elders [36]. Namely, a certain intensity of PA with a long duration (even years) is needed before cognitive changes can be observed [36]. While patients with moderate-intensity PA seemed to have the best cognitive performance at the 18-month follow-up interview and the greatest improvement in verbal fluency from the 18- to 36-month follow-up interview in our study, none of the associations was statistically significant after being corrected for multiple comparisons. It is worth noting that only 26.2% of our participants reported moderate and above intensity of PA at 6 months after diagnosis (data not shown), and this proportion doubled (53.2%) at 18 months after diagnosis. It is most likely that our participants substantially decreased their PA intensity due to cancer treatments at 6 months after diagnosis, and then returned to their normal exercises over time. By the time we assessed their cognitive functions, the majority of our participants might not have engaged in moderate and above intensity PA long enough after their cancer treatments, so that the beneficial effects of PA could not reach statistical significance in our study. Taken together, the decreased PA intensity due to cancer treatments may explain the non-significant associations between PA and cognitive recovery. Moreover, our data show that PA intensity increased by 1.6 MET-hours/week, while BMI decreased by 0.17 kg/m^2^ among our participants from 18 to 36 months after diagnosis (data not shown). Hence, exercise may have an indirect effect on cognitive change through modification of BMI or WHR. Future studies with a larger variation in PA and a longer duration of follow-up are warranted.

Strengths of this study include a population-based perspective longitudinal design and a large sample size. Furthermore, our findings regarding the association between normal WHR and verbal fluency recovery, as well as the association between normal BMI and memory recovery, indicated small to moderate clinical significance. A major limitation of this study is the absence of a cognitive assessment at the cessation of cancer therapy. The cognitive changes from the cessation of cancer therapy to 36 months after diagnosis should be more sizable than what we observed from 18 to 36 months after diagnosis. Therefore, the associations of age, BMI, WHR and PA with cognitive recovery and their clinical significance are probably underestimated in our study. Another limitation is that it was unclear whether our participants’ weight changed after breast cancer diagnosis and/or therapy, and if so, towards what direction. This potential recent weight gain/ loss may have also affected the outcome but could not be accounted for.

In conclusion, this study examined the associations of age, BMI, WHR and PA with post-therapy cognitive recovery in BC survivors. We found that the post-therapy cognitive recovery differed by age and obesity status. Our findings indicate that obesity control may be beneficial for the post-therapy cognitive recovery in BC survivors. Further research, including intervention trials, is warranted to confirm the causal relationship between obesity and cognitive impairments.

## MATERIALS AND METHODS

### Participants

Participants of the current study came from the Shanghai Breast Cancer Survival Study (SBCSS), a large population-based cohort study of 5042 female BC survivors aged from 20 to 75 who were diagnosed with primary BC between March 2002 and April 2006 in Shanghai, China. The detailed method has been reported elsewhere [37]. In brief, a baseline survey utilizing a structured questionnaire covering demographic characteristics, anthropometry, cancer diagnosis, progression and treatment, PA and other lifestyle factors, was carried out at approximately 6 months after BC diagnosis. Three subsequent follow-up interviews were conducted at 18, 36, and 60 months after cancer diagnosis. Survival information of the participants who had lost contact in follow-up was also ascertained by regularly matching death certificates from the Shanghai Vital Statistics Registry against SBCSS database.

The Cognition Sub-study comprised two assessments of cognitive function that were integrated into the 18- and 36-month follow-up interviews of the SBCSS. When the Cognition Sub-study was initiated, two thirds of the SBCSS participants had completed their 18-month follow-up interviews. As a result, 1507 patients who were alive at the 18-month follow-up interview with a diagnosis between December 2004 and April 2006 were approached for the Cognition Sub-study. Among them, 15 were excluded due to a prior history of other cancers; 62 were excluded because of BC *in situ*; and 45 were excluded because of a prior history of stroke; leaving a total of 1385 BC survivors eligible for the Cognition Sub-study. Characteristics of eligible participants of the Cognition Sub-study and participants of the SBCSS were compared, and no difference was found in demographics, age at cancer diagnosis, and clinical features between these two study populations [4]. Among the eligible participants, 1286 completed the first cognitive assessment (participation rate: 92.9%). Only 1143 patients were still alive at the 36-month follow-up interview and 1047 of them completed the second cognitive assessment (participation rate: 91.6%). This study was approved by the Shanghai Municipal Center for Disease Control and Prevention Ethical Review Committee and Vanderbilt University Institutional Review Board. All participants provided written informed consent.

### Cognitive function assessments

Participants’ cognitive function was assessed by trained interviewers using a comprehensive battery of cognitive tests, including: (1) the Logical Memory Subtest of the Chinese Version of Wechsler Memory Scale to measure immediate memory and delayed memory [38]; (2) the Chinese Version of Category Fluency Test to measure verbal fluency [39]; and (3) the Chinese Version of the Stroop Test to measure attention [40]. The diagnostic validity of this battery of cognitive tests was previously evaluated in Shanghai. We found it was a sensitive battery to discriminate Alzheimer's disease and mild cognitive impairment from healthy controls [4]. All interviewers were formally trained by a neurologist from Hua Shan Hospital, Shanghai, China.

### Exposures

Data on anthropometry and self-report PA collected in the SBCSS were used in the study. Participants’ weight and self-report PA were collected at the 6- and 18-month follow-up interviews, whereas height, waist circumference and hip circumference were measured only at the 6-month follow-up interview. BMI was calculated by dividing the body weight (in kilograms) by the height (in meters) squared (weight/height^2^), and was further classified into four categories according to the obesity classification for Chinese, including underweight ( < 18.5 kg/m^2^), normal (18.5-23.9 kg/m^2^), overweight (24.0-27.9 kg/m^2^) and obese ( ≥ 28.0 kg/m^2^) [41]. WHR was calculated as waist measurement divided by hip measurement, and was further divided into three categories according to the cut-off points recommended by WHO (0.85) and studies in Asian populations (0.80) for increased risk of metabolic complications, including normal ( < 0.80), 0.80-0.84 and abdominally obese ( ≥ 0.85) [42]. Levels of PA were estimated using metabolic equivalent-hours per week (METs-hours/week), which expresses the intensity and energy expenditure of activities in a way comparable among persons of different weight [43]. 1 MET (1 kcal/kg/hour) is roughly equivalent to the energy cost of sitting quietly at a metabolic rate of consuming 3.5 ml O^2^/kg/minute [44]. Intensity of PA was further divided into three categories according to the WHO recommendations on PA for adults, including light-intensity ( < 8.3 MET-hours/week), moderate-intensity (8.3-16.5 MET-hours/week), and vigorous-intensity ( ≥ 16.6 MET-hours/week) [45].

### Statistical analysis

Demographic variables and selected characteristics were compared between the subjects eligible for Cognition Sub-study and those who completed the study, using Student's *t*-test for continuous variables and Chi-square test for categorical variables. Linear regression models were used to examine the associations of age, BMI, WHR and PA with mean changes in cognitive scores of the period from 18- to 36-month follow-up interview (longitudinally), and with mean cognitive scores at 18-month follow-up interview (cross-sectionally). Bonferroni correction was used to control multiple comparisons. BMI and PA at 18-month follow-up interview were used in these analyses. Since waist and hip circumferences was not measured at 18-month follow-up interview, WHR at 6-month follow-up interview was used instead.

Linear regression analyses were adjusted for educational attainment, income, menopausal status, depression score, TNM stage, BC recurrence or diagnosis of other malignancies after the diagnosis of BC, cancer treatments (including surgery, radiotherapy, chemotherapy and tamoxifen use), and time since the cessation of cancer therapy. To control regression to the mean, analyses of longitudinal changes in cognitive scores of the period from 18- to 36-month follow-up interviews were additionally adjusted for cognitive scores at 18-month follow-up interview. We also evaluated the clinically meaningful importance of the longitudinal changes in cognitive function using effect size (i.e. Cohen's *d*: mean change in cognitive scores between two interviews divided by the standard deviation of cognitive score at the first interview) [46]. A Cohen's *d* of 0.2 is considered a small effect, 0.5 a moderate effect, and 0.8 a large effect. Cohen's *d* in the range of 0.2 to 0.3 is considered the minimally important difference for clinically meaningful effects [47]. Furthermore, we conducted sensitivity analyses limited to patients who underwent chemotherapy and additional analyses adjusted for comorbidities. All analyses were performed using SAS version 9.2 (SAS Institute, Cary, NC). Statistical significance was based on a two-sided probability with a significance level of 0.05.
